# Isolation of Murine Myeloid Progenitor Populations by CD34/CD150 Surface Markers

**DOI:** 10.3390/cells11030350

**Published:** 2022-01-20

**Authors:** Leonid Olender, Roshina Thapa, Roi Gazit

**Affiliations:** Shraga Segal Department of Microbiology, Immunology, and Genetics, Faculty of Health Sciences and the National Institute of Biotechnology in the Negev, Ben-Gurion University of the Negev, Beer-Sheva 84105, Israel; olender@post.bgu.ac.il (L.O.); thapa@post.bgu.ac.il (R.T.)

**Keywords:** myeloid progenitors, murine bone marrow, cell sorting, hematopoiesis

## Abstract

Myeloid progenitors are intermediates between Hematopoietic Stem Cells (HSCs) and Myeloid effector progeny. In mouse bone marrow, they are part of the Lineage^−^ cKit^+^ Sca1^−^ (LK) compartment. To date, most researchers used CD34 and FcγR surface markers for the dissection of this compartment into various populations. Surprisingly, however, this approach does not provide distinct separation by fluorescence-activated cell sorting (FACS). In this study, we suggest using CD150 instead of FcγR. We re-analyzed published single-cell RNA-Seq data and found that CD34/CD150 provides better sub-populations separation, compared to the “classical” CD34/FcγR-based approach. We confirm our findings by independent FACS analysis. We demonstrate comparable differentiation potential of the newly-obtained LK sub-populations, like previous “classical” ones. Therefore, we suggest the CD34/CD150 gating strategy, utilizing commonly-used surface markers, as a robust and reproducible separation of the LK compartment into distinct sub-populations.

## 1. Introduction

Blood and immune cells are constantly generated in the bone marrow (BM). Progenitors are defined as short-lived intermediates between Hematopoietic Stem Cells (HSCs) and effector cells [1]. Surprisingly, however, the identification and isolation of hematopoietic progenitors are technically limited. Recently, single-cell RNA-seq suggested an updated view on the hierarchy of hematopoiesis [2,3,4,5]. Nevertheless, there is still a need for a relatively simple way for flow cytometry-based identification and isolation of distinct hematopoietic progenitor populations.

Hematopoietic progenitors were initially considered a byproduct population obtained during the isolation of HSCs. The seminal study of Akashi and Weissman demonstrated that the Lin^−^ cKit^+^ Sca1^−^ (LK) population could be further dissected by the surface expression of CD34 and FcγR (CD16/32) markers [6]. Using ex-vivo assays, they found that cells with the lowest CD34/FcγR had a Megakaryocyte/Erythrocyte potential. The CD34^+^FcγR^hi^ cells had a Granulocyte/Monocyte potential, and CD34^+^FcγR^lo^ cells had mixed potential and gave rise to the various types of myeloid cells. This scheme, calling for Megakaryocyte/Erythrocyte (MEP), Granulocyte/Macrophage (GMP), and the Common-Myeloid Progenitors (CMP), had become the canonical dogma [7,8,9,10]. Following studies had suggested more sophisticated schemes. For example, Pronk et al., showed that the LK compartment could be dissected into a number of functionally distinct sub-sets by surface expression of Endoglin (CD105), CD41, CD150, and Ter-119 [11]. This brilliant study used an elaborated multi-color panel, which is not used often.

A number of studies published in recent years have claimed that phenotypic CMPs might not be an actual population [12,13,14]. However, isolation of various myeloid progenitor sub-populations is problematic not only because of that, but, more basically, because there is not an easy way to determine borders between the putative MEPs, CMPs, and GMPs [6]. This is not a concern within one study but might be an obstacle for meta-analysis of independent studies. Here we demonstrate that, by using the CD34 and CD150 surface markers, we were able to divide the LK compartment into three distinct sub-populations of CD34^−^CD150^−^, CD34^mid^CD150^+^, and CD34^+^CD150^−^ (which we called sMEP, sCMP, and sGMP, respectively, in contrast to cCMP, cGMP and cMEP gated by CD34/FcγR). We further characterize the potential of the newly-obtained populations and compare them to the “classical” ones, using short-term transplantations, liquid cultures, and methylcellulose colony-forming assays. This novel approach relies on markers commonly used by hematopoiesis researchers and might help improve the reproducibility of independent studies.

## 2. Materials and Methods

### 2.1. Single-Cell Data Analysis

Gene expression and index–sort data were adapted from Nestorowa et al. (Nestorowa et al., 2016). We used the free Flowing Software (https://bioscience.fi/services/cell-imaging/flowing-software/, developed by Perttu Terho from the Cell Imaging Core of the Turku Centre for Biotechnology, Turku (Finland)) to re-analyze and visualize the index-sort data. The software was downloaded 13.02.20 and last accessed 29.07.2021. The single-cell RNA-seq. data referring to the expression of 4282 genes with a squared coefficient of variation exceeding the technical noise was visualized using the principal component analysis (PCA) by MATLAB code adopted from Cai et al. [15].

### 2.2. Bone-Marrow Cells Extraction

Femur, tibia, and pelvic bones were collected from C57BL/6J mice, crushed in Sample Media (PBS, 2 mM EDTA, 2% FBS), and enriched for mononuclear cells using Histopaque1083 separation reagent (Sigma-Aldrich, Rehovot, Israel). All experiments were carried out according to the ethical guidelines following the approval of the Ben-Gurion University and the Israel Animal Care and Use Committees. Protocols and dates of approval: IL-68-11-2028D (24.01.2019) and IL-06-02-2021C (20.05.2021).

### 2.3. Staining and Cell-Sorting

BM mononuclear cells were stained on ice in 50 µL volume with a mixture of labeled antibodies for 90 min. Cell-sorting and flow-cytometric analysis were performed using the FACS-Aria III device (BD). Specific antibodies from Biolegend (San Diego, CA, USA): Lineage cocktail-Pacific Blue, cKit-APC-Cy7, Sca1-APC, CD45.2–Pacific Blue, Gr-1 (Ly-6G/Ly-6C)-FITC, Ter119-PerCP/Cy 5.5, CD41-PE and CD150-PE-Cy7; eBiosccience (San Diego, CA, USA): CD34-FITC, and FcγR-PE; TONBO Biosciences (San Diego, CA, USA): CD45.1-APC and Mac-1 (CD11b)-PE-Cy7.

### 2.4. Transplantation Assay

Myeloid progenitors sorted from CD45.1 donors were injected into tail veins of non-irradiated CD45.2 recipients. Seven days later, spleens and bone marrows were extracted, and depletion of erythrocytes was performed using ACK red blood cell lysis buffer (8.29 g/L NH_4_Cl, 1 g/L KHCO_3_, and 0.1 mM EDTA, pH 7.2–7.4). In brief, cells were homogenized, washed in sample medium, and incubated in ACK buffer (5 mL per sample) for 5 min. Then they were washed in PBSX1 and stained with Mac-1-PC7 antibody and DAPI and analyzed using BD-FACS Aria III cell sorter.

### 2.5. Liquid Cell Culture and Giemsa Staining

Sorted cells were cultured in Biotarget medium, supplemented with 10% fetal bovine serum, 2% Pen-Strep solution, 2% L-Glutamine solution (all from (Biological Industries, Beit-Haemek, Israel), 20 ng/mL SCF, 10 ng/mL Flt3L, 20 ng/mL IL11, 10 ng/mL IL3, 10 ng/mL GM-CSF, 10 ng/mL TPO and 6 ng/mL EPO (all from PeproTech, Rehovot, Israel). For Giemsa staining, cells were cyto-spined onto slides, fixed in methanol absolute for 30 s, washed in double-distilled water (DDW), incubated in Giemsa solution (Fluka Analytical, Buchs, Switzerland, diluted 1:20 with DDW), dried and covered.

### 2.6. Colony-Forming Assay

Five thousand freshly sorted cells were plated in 0.5 mL of semi-solid methylcellulose medium (#HSC006, RnD systems) per well in a 24-well plate. We added 20 ng/mL SCF, 10 ng/mL Flt3L, 20 ng/mL IL11, 10 ng/mL IL3, 10 ng/mL GM-CSF, 10 ng/mL TPO and 6 ng/mL EPO (all from PeproTech, Rehovot, Israel), and Pen-Strep solution (Biological Industries, Beit-Haemek, Israel) at the final concentration of 2%. Plates were incubated at 37 °C, 5% CO_2_ and assessed for colonies seven days later.

## 3. Results

### 3.1. CD150 Might Substitute FcγR for FACS Dissection of the LK Compartment

**Figure 1:**
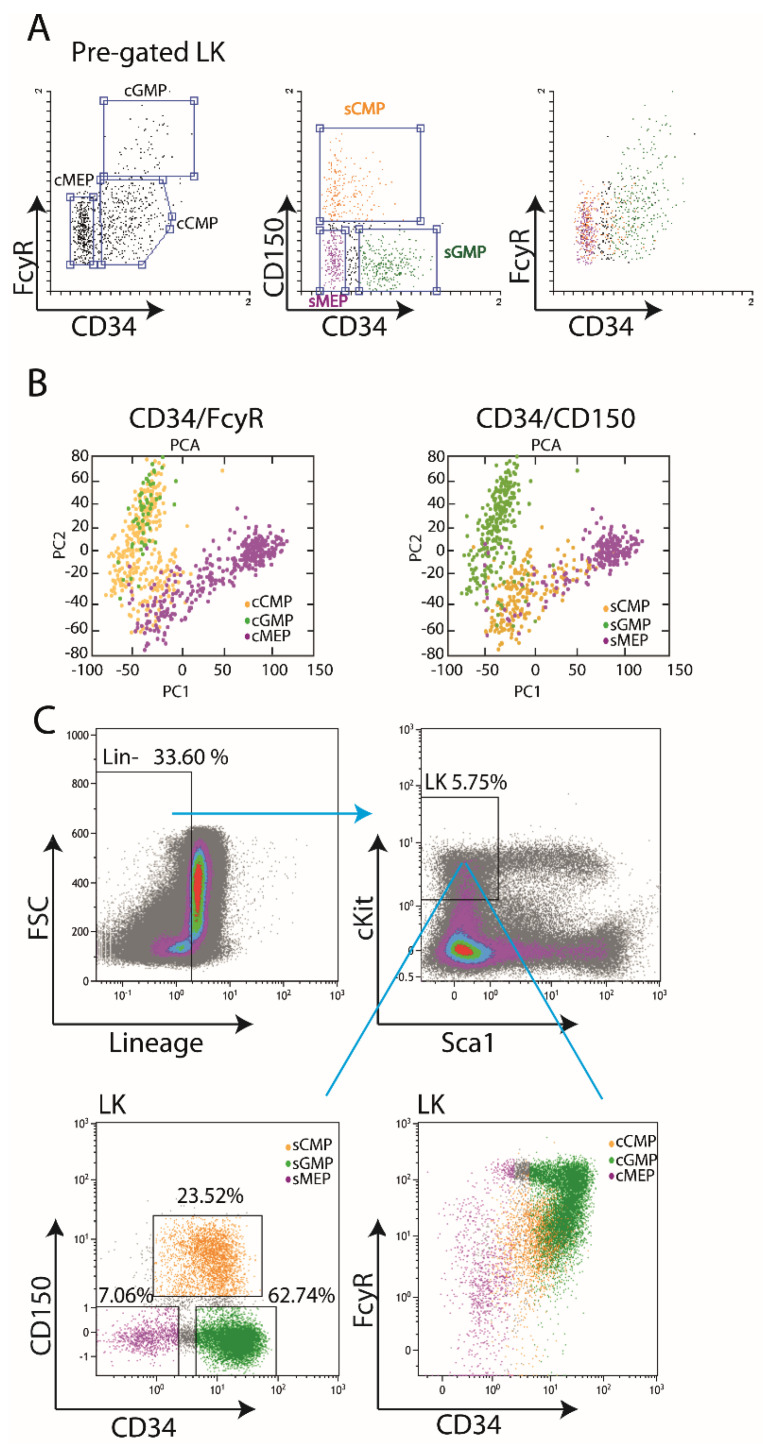
Surface expression of CD34/CD150 suggests better separation of the LK compartment compared to CD34/ FcγR. (**A**) Index-sort data from Nestorowa et al., was re-analyzed and visualized (see Materials and methods). LK compartment was dissected using the classical CD34/FcγR (left plot) or CD34/CD150 approach (center plot)**.** Right plot shows the CD34/CD150 sub-populations (sCMP Orange, sGMP Green, and sMEP Pink) on a CD34/FcγR plot. (**B**) Principal component analysis (PCA) score plot of 4282 variable genes within the myeloid progenitor LK compartment from Nestorowa et al., gated either by CD34/FcγR (left plot) or by CD34/CD150 (right plot). PCA plot of the single-cell data characterizes the trends exhibited by the expression profiles of CMPs (orange), GMPs (green), and MEPs (pink). Each dot represents a single cell, and each color represents the gated subpopulation based on CD34/FcγR (left) or CD34/CD150 (right). Numbers of cells within each population were as follows: cCMP: 332, cGMP: 55, cMEP: 339, sCMP: 200, sGMP: 278 and sMEP: 211. (**C**) Fresh BM mononuclear cells shown with the dissection of LK cells either by CD34/CD150 (left), or by CD34/FcγR (right), colors as above.

Practical FACS dissection of the Lineage^−^cKit^+^Sca1^−^ (LK) cell compartment using the “classical” CD34 /FcγR strategy presents a continuum, not clearly separated sub-populations. We considered using alternative surface markers. Of the common surface markers for mouse stem- and progenitor cells, CD150 is differentially expressed on LK cells. We utilized Nestorowa et al. [16].data, which included single-cell RNA-Seq data and immune-phenotype. We re-analyzed the published index-sort surface marker expression data and compared CD34/CD150 or CD34/FcγR gating using the Flowing software (see Section 2). As shown in Figure 1A, visualization of the index-sort data showed that the CD34/CD150 might yield better-separated sub-populations than the CD34/FcγR. Next, we performed Principal Component Analysis (PCA) to compare gene expression profiles of the sub-populations obtained by either approach (Figure 1B). PCA revealed a more distinct separation between the CD34/CD150 sub-populations (sCMP, sGMP, and sMEP) than between the CD34/FcγR sub-populations (cCMP, cGMP, and cMEP, Figure 1B), supporting the CD34/CD150 strategy. Furthermore, our independent flow-cytometric analysis demonstrated a better separation when using the CD34/CD150 than CD34/FcγR (Figure 1C). Taken together, these data suggest the advantage of the CD34/CD150 strategy, compared to CD34/FcγR, for the dissection of the LK compartment into separated sub-populations using flow cytometry.

### 3.2. Differentiation Potential of CD34/CD150 Sub-Populations Is Comparable to That of CD34/FcγR Ex-Vivo

**Figure 2:**
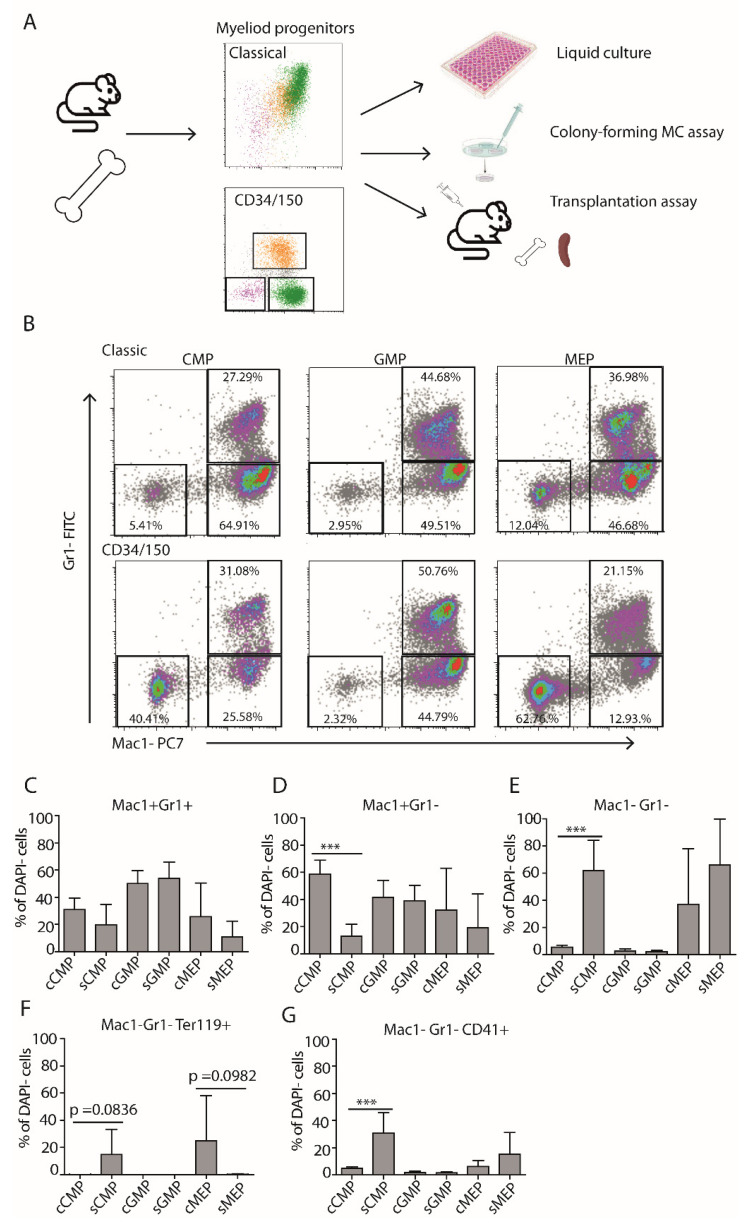
Comparable differentiation potential of CD34/FcγR and CD34/CD150 myeloid progenitor sub-populations in culture. Myeloid progenitor sub-populations were sorted either by CD34/FcγR or by CD34/CD150, plated and cultured for eight days. (**A**) Schematic representation of the experimental procedure. (**B**) Representative FACS plots showing surface expression of Mac-1 and Gr-1 in cultures derived from the defined sub-populations at day eight in culture. (**C**–**G**) Frequencies of the differentiated cell types in culture. Data from *n* = 4 independent experiments. Statistical significance was calculated by t-test, *** *p* ≤ 0.001.

Next, we wanted to assess the differentiation potential of the newly obtained LK sub-populations and compare it to the “classical” CD34/ FcγR counterparts. We aimed to examine multiple assays, including liquid culture, methylcellulose colony-forming assay, and transplantation (Figure 2A). First, we grew cells sorted from all six sub-populations in medium supplemented with serum and cytokine combination, supporting various myeloid lineages’ differentiation (see Materials and Methods). Interestingly, sCMPs produced significantly more Mac-1^−^ Gr-1^−^ and significantly fewer Mac-1^+^ Gr-1^−^ cells than cCMPs (Figure 2D,E). sCMPs also exhibited higher erythroid and megakaryocyte differentiation potential (defined here as frequencies of Mac-1^−^ Ter-119^+^ and Mac-1^−^ CD41^+^ cells, respectively) compared to cCMPs, even if not significantly different in part of the parameters (Supplementary Figure S1 and Figure 2F,G). The cGMPs and sGMPs were fairly similar, showing higher potential for the production of granulocytes (defined here as Mac-1^+^ Gr-1^+^ cells) compared to any of the CMP or MEP sub-populations (Figure 2B,C). Finally, cMEPs and sMEP tended to produce less Mac-1^+^ and more erythroid/megakaryocyte progeny (Supplementary Figure S1 and Figure 2F,G).

**Figure 3:**
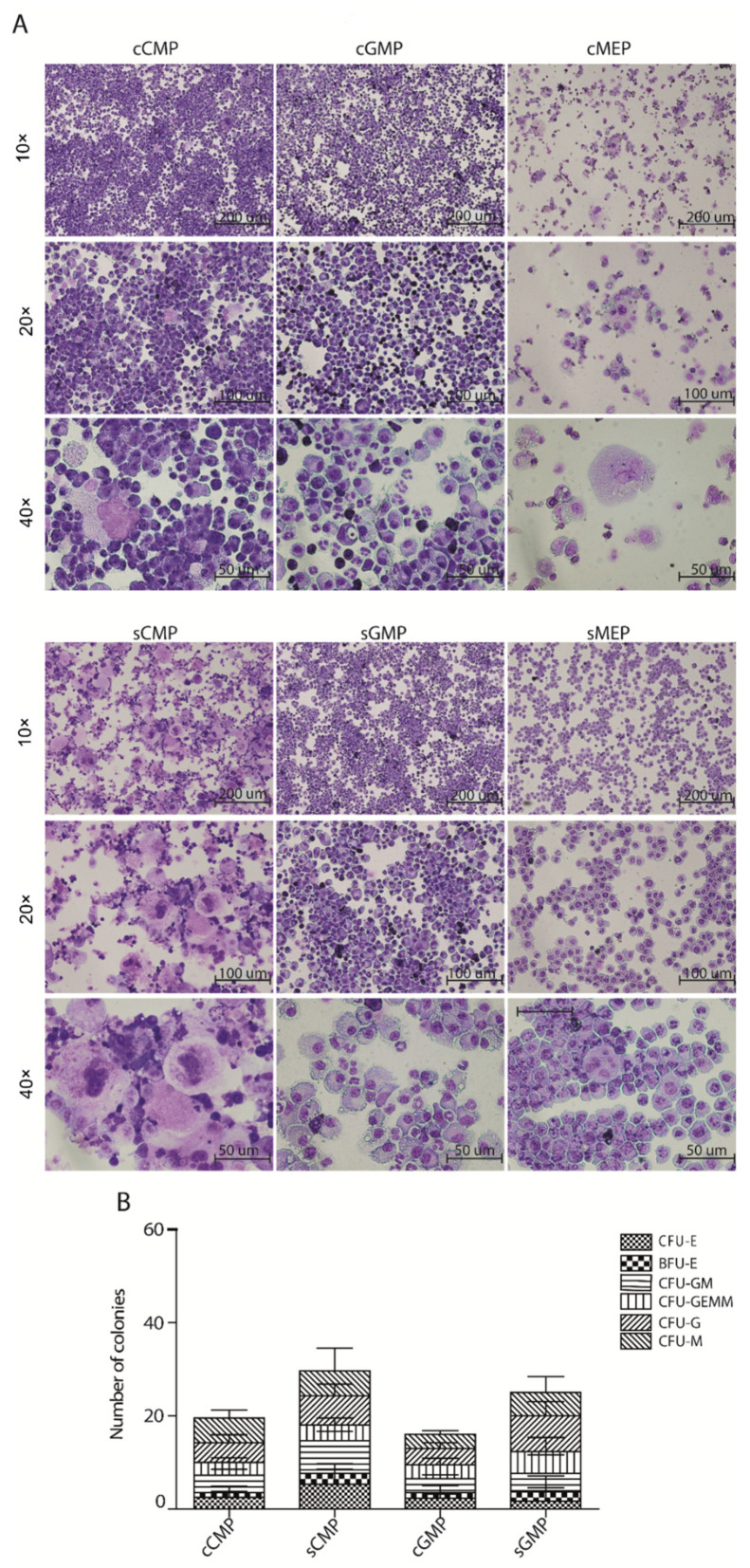
Morphological assessment of CD34/FcγR like CD34/CD150 sub-populations finds comparable phenotype ex vivo. (**A**) LK cells sorted by CD34/FcγR or by CD34/CD150. Giemsa staining of cells derived from defined sub-populations after eight days in culture. Scale bar = 200, 100, and 50 µm for the 10×, 20×, and 40×, respectively. (**B**) Cells sorted as before were plated in semi-solid methylcellulose and cultured for seven days. Graphs represent counts of colonies of various types. Summary of data from *n* = 2 independent experiments with a total of *n* ≥ 3 technical replicas per population.

In addition to the immune-phenotype, we performed Giemsa staining to visualize cellular morphology, revealing more megakaryocyte giant cells in sCMP-derived cultures than cCMPs, in line with the flow-cytometric data (Figure 3A). The cGMP and sGMP showed less variable Giemsa, with plenty of Granulocytes and Monocyte/Macrophages (Figure 3A). Giemsa slides prepared from cMEP or sMEP showed less clearly defined differentiated cells, with no major differences between them (Figure 3A). Thus, liquid cultures show that either CD34/CD150 or CD34/FcγR fractionate the LK compartment into sub-populations of mixed-lineage potency. The cCMP/sCMP, cGMP/sGMP and cMEP/sMEP are largely comparable.

In parallel, we performed a methylcellulose colony-forming assay with a similar combination of cytokines (Figure 3B and Supplementary Figure S2). However, cMEPs and sMEPs did not yield colonies (data not shown). While sCMPs and sGMPs produced slightly more colonies than their respective CD34/FcγR counterparts, we did not observe significant differences between the four populations in terms of colony types (Figure 3B). Taken together, our ex-vivo data suggest slight differences but primarily a comparable differentiation potential of the LK sub-populations sorted by CD34/CD150 or by CD34/FcγR.

### 3.3. Transplantation of CD34/CD150 or CD34/FcγR Sub-Populations Yields Comparable Progeny In Vivo

**Figure 4:**
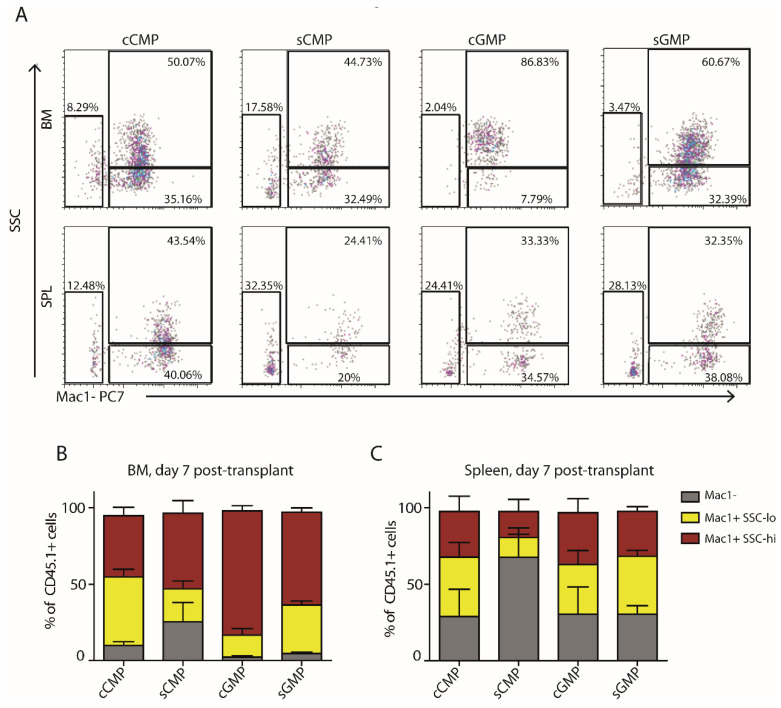
Transplantation of CD34/CD150 or CD34/FcγR sub-populations yields comparable progeny in vivo. Myeloid progenitor sub-populations sorted from CD45.1 donors by CD34/FcγR or by CD34/CD150 and transplanted into non-irradiated CD45.2 congenic recipients. Spleens and BM from tibiae and femur were harvested seven days later, and FACS analyzed. (**A**) Representative plots showing surface expression of Mac-1/Side-Scatter (SSC) of CD45.1 donor-derived cells. (**B**,**C**) Histograms summarizing frequencies of the differentiated myeloid progeny in the BM (**B**) and spleen (**C**). Graphs represent data from *n* = 4 independent experiments.

We also wanted to test the potency of the LK sub-populations in vivo. For this purpose, we performed transplantations of CD45.1 donors into non-irradiated CD45.2 congenic hosts, following a recently published study [17] (Figure 4 and Supplementary Figure S3). Importantly, we did try transplantation into lethally irradiated recipients. Still, in agreement with Yanez et al., we observed a significant difficulty with this extreme perturbation that induces severe inflammation in the recipient mice. We extracted spleens and BM from recipients seven days after transplantation and stained them for lineage markers (Mac-1, CD41, and Ter-119). Transplantation of either cMEPs or sMEPs did not yield sufficient CD45.1^+^ progeny for analysis (data not shown). The recipients of either cCMP, cGMP, sCMP, or sGMP showed mixed donor-derived progeny with some differences in their relative frequencies (Figure 4A–C). sCMPs produced slightly more Mac-1^−^ progeny in the BM and the spleen (Figure 4B,C). Both cGMPs and sGMPs produced a little more Mac-1^+^ SSC^hi^ granulocytes than their respective CMPs in the BM (Figure 4B). Dissection of the BM Mac-1^−^ population by CD41 and Ter-119 suggested that CMPs sorted by either approach had comparable erythroid/megakaryocyte potential higher than that of their respective GMP counterparts (Supplementary Figure S3). In the spleen, the cGMPs seemed to produce more erythroid/megakaryocyte progeny than the sGMP, but the difference was not significant, cCMP and sCMP were again comparable (Supplementary Figure S3). Taken together, these data suggest comparable differentiation potency of either CD34/CD150 or CD34/FcγR sub-populations of the LK compartment upon transplantation into non-irradiated hosts.

## 4. Discussion

Flow-cytometry sorting of hematopoietic stem- or progenitor cells provided a major leap forward for science. Various strategies, using multiple surface-markers, advanced the field. Here we present the option to dissect the LK compartment using CD34/CD150 instead of CD34/FcγR, obtaining better separation on FACS plots and comparable differentiation potency of the myeloid progenitor’s sub-populations. This will help multiple laboratories to improve reproducibility and robustness.

Pioneer studies of the hematopoietic system suggested for sequential differentiation cascade from the primitive multipotent HSCs down to terminally-differentiated effector cells [6,11]. HSCs possess life-long self-renewal capacity, which diminishes in their proximal Multipotent Progenitors (MPPs) and is probably lost in the lineage-committed progenitors such as CMPs [18,19]. In recent years, differentiation paths were expanded with discoveries of “shunts,” allowing rapid direct differentiation from HSCs into Megakaryocytes [2,11,20]. Emerging new technologies of single-cell RNA sequencing provided new opportunities for the molecular study of stem- or progenitor cells. Some of these studies reported that “classical” CMPs might not exist as a “pure” population since they could not identify cells with multi-lineage transcription factor repertoire within the LK CD34^+^FcγR^lo^ sub-population [13,14]. Such studies allowed the scientific community to revisit the dogma of the hierarchical hematopoietic tree and redraw some of its branches. Recently, a new technique has been introduced to investigate the dynamics of CMPs transcriptome further, making the concept of bi-potency part of scientific discussion again [4]. The introduction of alternative markers for separation between the sub-populations of myeloid progenitors, such as CD34/CD150 presented here, can help to resolve controversies and gain robust reproducibility. We are aware that the absence of methylcellulose assay and in vivo data for MEP populations, possibly due to technical issues, does not allow us to reach conclusions regarding these populations’ potential.

The CD34/CD150 dissection of the LK compartment provides better separation into distinct sub-populations. Re-analysis of scRNA-Seq data also suggest better separation of transcriptome profiles. Independent FACS analysis shows a much better resolution between the sub-populations. Notably, the expression of surface markers does not necessarily match gene expression at a single-cell level, reflecting potential heterogeneity within the sub-populations. Our study may provide better reproducibility and robustness for analysis and cell sorting among independent laboratories. Improving techniques for identifying and isolating putative myeloid progenitors will help expand our understanding of hematopoiesis dynamics.

## Supplementary Materials

The following supporting information can be downloaded at: www.mdpi.com/article/10.3390/cells11030350/s1, Figure S1: Data related to Figure 2; Figure S2: Data related to Figure 3; Figure S3: Data related to Figure 4.
