# Supplementary material for: Isolation of Murine Myeloid Progenitor Populations by CD34/CD150 Surface Markers
Source: Cells. 2022 Jan 20;11(3):350. doi: 10.3390/cells11030350 (PMC8834359; doi:10.3390/cells11030350)
Supplement: Supplementary file 1 [file cells-11-00350-s001.zip › cells-1553174-supplementary.pdf]

Supplementary information for:

Isolation of Murine Myeloid Progenitor Populations by CD34/CD150 Surface Markers

Authors: Leonid Olender [a,*], Roshina Thapa [a,*], Roi Gazit [a,§]

Affiliations: [a] The Shraga Segal Department for Microbiology, Immunology, and Genetics; Faculty of Health Sciences; National Institute for Biotechnology in the Negev; the Ben-Gurion University of the Negev, Israel POB 84105

* These authors contributed equally to this work
[§] Correspondence to gazitroi@bgu.ac.il

Three figures with caption

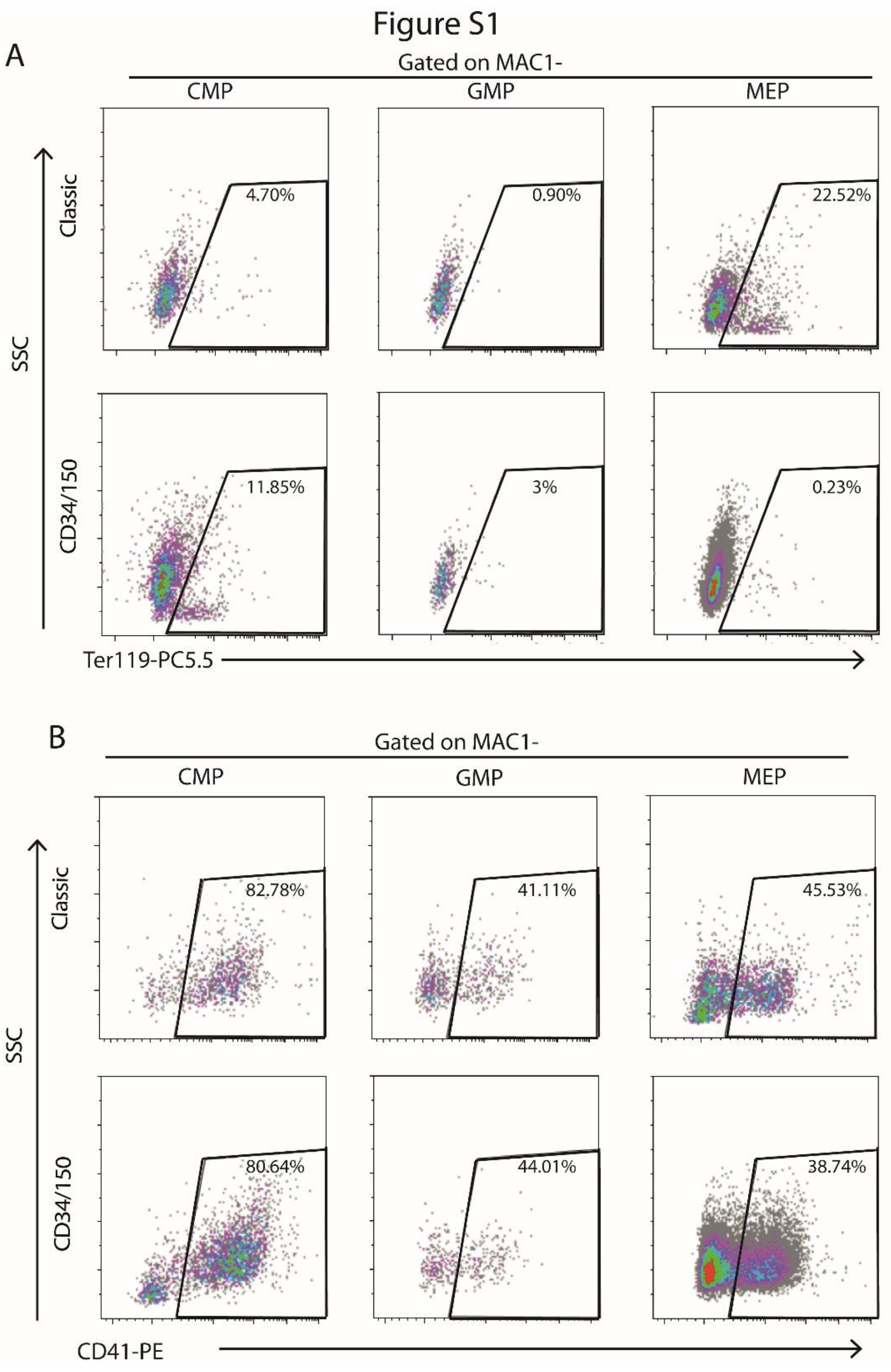

Supplementary Figure S1. Data related to Figure 2; Comparable Erythroid/ Megakar-yocyte differentiation of CD34/FcγR and CD34/CD150 sub-populations in culture. Representative FACS plots of Ter-119 (**A**) or CD41 (**B**) surface expression on cells after 8 days in culture.

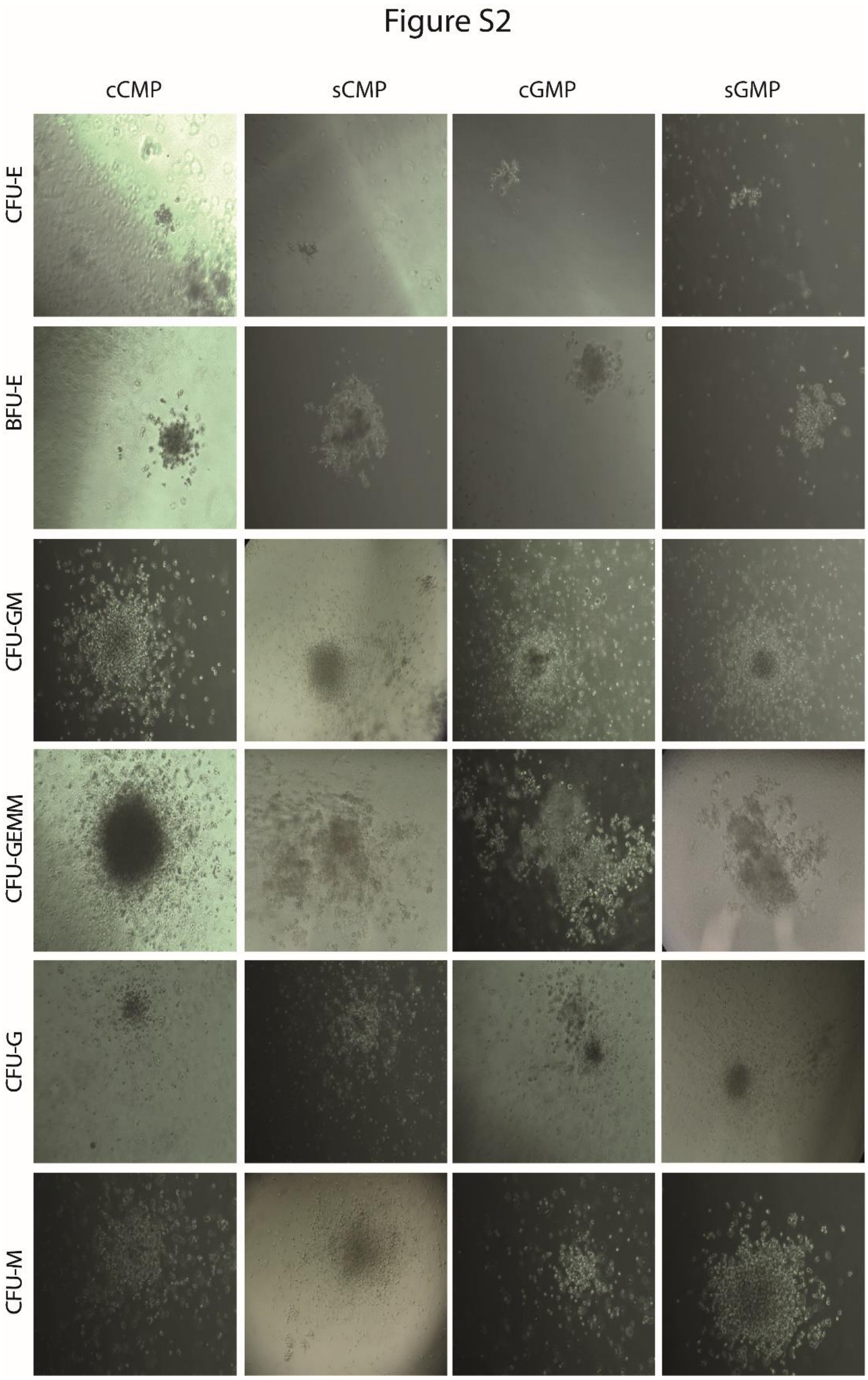

Supplementary Figure S2. Data related to Figure 3. Representative images of methyl-cellulose colonies. Cells sorted as described in the main text, plated in semi-solid methylcellulose and cultured for 7 days. Pictures taken with 20X magnification.

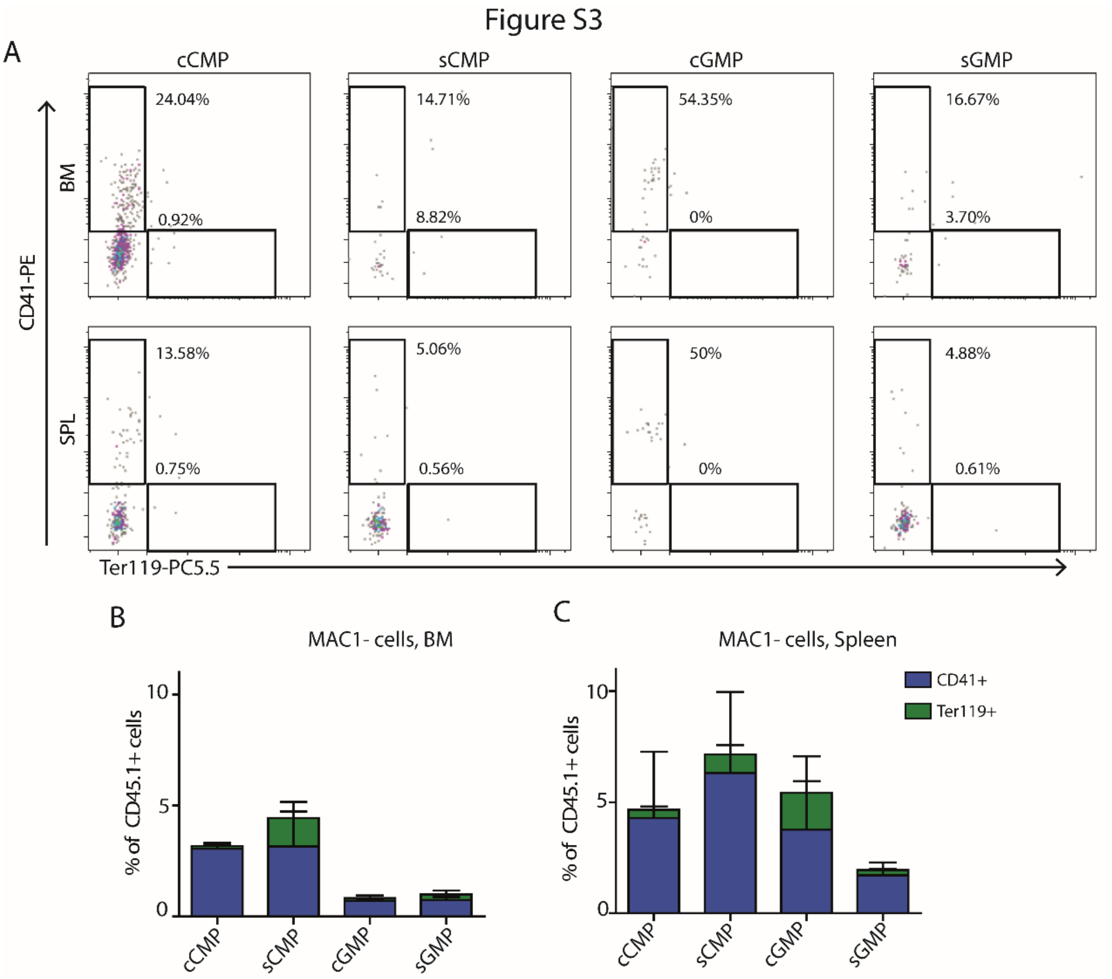

Supplementary Figure S3. Data related to Figure 4 Transplantation of CD34/CD150 or CD34/FcγR sub-populations yields comparable progeny in vivo. Representative FACS plots (**A**) and graphs (**B**,**C**) showing surface expression of CD41 and Ter-119 in donor-derived Mac-1 negative cells from the CD45.1 donors. Bone marrow and spleen data are shown. Graphs represent data from n = 4 independent experiments.
